# Disentangling depression in Belgian higher education students amidst the first COVID-19 lockdown (April-May 2020)

**DOI:** 10.1186/s13690-020-00522-y

**Published:** 2021-01-07

**Authors:** Jeroen De Man, Veerle Buffel, Sarah van de Velde, Piet Bracke, Guido F. Van Hal, Edwin Wouters, for the Belgian COVID-19 International Student Well-being Study (C19 ISWS) team, Sylvie Gadeyne, Hanne P. J. Kindermans, Mathilde Joos, Sander Vanmaercke, Vlaamse Vereniging van Studenten, Anne-Sophie Nyssen, Ninon Puttaert, Dries Vervecken, Marlies Van Guyse

**Affiliations:** 1 Department of Family Medicine and Population Health, University of Antwerp, Doornstraat 331, 2610 Wilrijk, Belgium; 2 Department of Sociology, University of Antwerp, Antwerp, Belgium; 3 Department of Sociology, Ghent University, Antwerp, Belgium; 4 Social Epidemiology and Health Policy, University of Antwerp, Antwerp, Belgium

**Keywords:** COVID-19, Stay-at-home order, Belgium, Mental health, Higher education students, Depressive symptoms, Academic stress, Fear of infection, Institutional dissatisfaction

## Abstract

**Background:**

The surge of COVID-19 infections has prompted many countries to take unprecedented policy measures. In Belgium, the authorities implemented a nation-wide stay-at-home order for several months. Evidence of the mental health effect of such measures is scarce. A recent review by Brooks et al. has compiled a defined list of stressors affecting people’s mental health under quarantine during previous epidemic settings. This study aims to test the association between these stressors and the mental health of students attending higher education during the stay-at-home order in Belgium.

**Methods:**

In this cross-sectional study, 18,301 students from 13 higher education institutions (HEI) participated in an online survey between 26 April and 11 May 2020. We assessed the association between potential stressors and depressive symptoms severity scores and structural equation modeling was used to assess how stressors may mediate the association between duration of exposure and depressive symptoms severity.

**Results:**

The stressors proposed by Brooks et al. were found to be associated with depressive symptoms severity. The stressors ‘perceived academic stress’, ‘institutional dissatisfaction’ and ‘fear of being infected’ were associated with substantially higher depressive symptoms severity scores. The association between duration of exposure and depressive symptoms severity was mediated by ‘academic stress’. Being in a steady relationship and living together with others were both associated with a lower depressive symptoms severity.

**Conclusion:**

Findings underline the need for a student-centered approach and mental health prevention. Authorities and HEIs should consider *whether* and if so, *how* a stay-at-home order should be implemented.

**Supplementary Information:**

The online version contains supplementary material available at 10.1186/s13690-020-00522-y.

## Background

The COVID-19 pandemic has had a profound impact on global health, with, as of September 2020, more than 27 million confirmed cases and almost 900,000 deaths [1]. Belgium was hit hard by the pandemic: by September 2020, authorities reported more than 88,000 confirmed cases and almost 10,000 deaths. Transmission of COVID-19 within the country was confirmed in early March 2020 and increased rapidly during March and April 2020. Belgium was among the world’s worst-affected countries in terms of the number of deaths per capita attributed to COVID-19, although this was likely due to using a different method of reporting compared to other countries [2].

The rapid spread of the virus spurred a series of unprecedented policy measures, including a stay-at-home order. On 18 March, strict physical distancing measures were imposed, with non-essential travel prohibited [3]. People were only allowed to leave their house to buy food or go to work if considered essential (e.g., people working in healthcare, transportation, food distribution, etc.). All bars, restaurants and shops providing non-essential services were closed, but individual physical exercising was still allowed. Gatherings were banned, and students were prohibited to attend classes physically and higher education institutions (HEI) were forced to move to online teaching methods. Penalties were imposed for individuals who failed to comply [3].

The implementation of such radical measures comes with multiple side-effects, and experts expressed their concern about a profound impact on mental health [4]. However, direct physical consequences (i.e., morbidity and mortality directly caused by COVID-19) have typically been given more weight in decision-making and public health interventions. An example of this was the suspension of schools in 188 countries, affecting over 90% of enrolled students globally (1.5 billion young people) [5], despite conflicting evidence of a substantial contribution to COVID-19’s transmission control [6]. To obtain a more balanced image, mental health experts have been calling for immediate and high-quality evidence on the mental health impact of the COVID-19 pandemic and related containment measures from novel population-based studies [4]. In addition, experts reached a consensus on the urgent need to identify the mechanisms that are affecting mental health during the pandemic in order to provide evidence-based and mechanistically informed psychological treatment and public health interventions [4].

College and university students have been shown a vulnerable group regarding mental health. Mental disorders have been shown common in this population and are typically untreated [7]. A German study reported a higher prevalence of depressive symptoms in university students compared to the general population, which the authors attributed to an increase in academic demand and a decrease in peer support [8]. During the COVID-19 pandemic, students have been confronted with a combination of government and institutional specific measures – potentially adding to their mental health vulnerability [5]: 1) a radical transformation from in-person to online teaching and evaluation; 2) cancellations of anticipated events such as exchange studies, internships, graduation ceremonies, etc.; 3) the loss of part-time jobs; and 4) a shrinking job market enhancing uncertainty among students in their final year.

Preliminary evidence from studies conducted during the current pandemic indeed seems to suggest their age group to be among the most affected [9–12]. Moreover, a study in 1000 university students in Greece reported an alarming increase in suicidal thoughts and symptoms severity scores of depression and anxiety during the first phase of a national stay-at-home order [13]. A study in 7143 medical students in China reported a high level of anxiety and found an association with COVID-19-related stressors such as economic stressors, effects on daily-life, and academic delays [14]. A study with university students in Spain also reported a negative effect on their mental health [15]. However, it remains unclear why and how this subgroups’ mental health has been affected, and several potential stressors remain unexplored.

A recent review identified a comprehensive set of mental health stressors that were shown to play a role in specific populations of infected people who were quarantined to limit the transmission of pathogens similar to COVID-19 [16]. These stressors included: frustration and boredom, inadequate supplies of resources, inadequate information from public health authorities, insufficient financial resources, perceived stigma, and fears of infection. Duration of the exposure to quarantine was also found to play a role, but it was unclear through which mechanism.

We hypothesize that the same type of mental health stressors, after contextualization, could play a role among students attending higher education exposed to a stay-at-home order during the COVID-19 pandemic. Contextualization of these stressors relates to the study population (i.e., higher education students) and to the exposure (i.e., a stay-at-home order vs. quarantine). For example, tailored to this study population of students, we propose to examine how COVID-19 related repercussions on their academic work (i.e., academic stress) and their institutions’ response to the COVID-19 pandemic (i.e., institutional dissatisfaction) are related to the mental health of these students. Of this compilation of stressors, we further hypothesize that the level of boredom and academic stress may typically worsen over a relatively short time period (i.e., 2 weeks). As such, we think that these two stressors may explain the assumed association between duration of exposure to the stay-at-home and mental health outcomes.

This study aims to investigate the association between the COVID-19 pandemic – and related containment measures – and students’ mental health attending higher education in Belgium. In particular, we will examine the association between depressive severity scores and a set of mental health stressors identified by Brooks et al. during quarantine, after tailoring them to our study population. Finally, we will test whether the association between the duration of the stay-at-home order and students’ mental health is mediated through the stressors’ perceived boredom’ and ‘perceived academic stress’.

## Methods

### Data-collection and procedures

In order to address the above-cited research aims, the study employed a structured questionnaire administred to a convencience sample of students: a link to an online survey was sent by e-mail to the students of 13 higher education institutions (HEI) located in Belgium. Participants were eligible if they were enrolled in a higher education program, aged 18 or above, and provided informed consent. Data collection took place between 26 and 04-2020 and 11-05-2020 corresponding to day 39 and 54 after the stay-at-home order started. Twenty-five thousand two hundred seventy-two students started the survey and 21,270 students completed it. The study population was further reduced to 18,301 after applying additional selection criteria: age below 31 years, enrolled in a bachelor’s or master’s program, residing in Belgium during the pandemic and not having been diagnosed with COVID-19 at the time of the study. The latter group was not included to avoid interference with the stressor ‘fear of infection’.

The study is part of the COVID-19 International Student Well-being Study (C19 ISWS) in which 110 HEIs of 26 countries participated. More details about the study procedures can be found in the study protocol [17].

The 8 items of the CES-D scale, used as the response variables, did not have missing values. The percentage of missing data in other variables varied between 0.0 and 0.5%, except for perceived stigma (6.1%). Multivariate imputation by chained equations with predictive mean matching for continuous data, logistic regression imputation for binary data and polytomous regression imputation for unordered categorical data (factor > 2 levels) was used to handle the missing data under a missing at random assumption. The procedure was done using the ‘Mice’ package in R [18]. Rubin’s rules were used to pool point and SE estimates across 20 imputed data sets. The results of this approach were compared with the results of a complete case analysis.

### Measures

Standard demographic and socioeconomic questions were asked to determine gender, age, the number of people living with, citizenship, relationship status, and financial ability to cover living expenses before the stay-at-home order.

The eight-item version of the Center for Epidemiological Studies Depression Scale (CES-D scale) [19] was used to measure severity of depressive symptoms and has shown adequate psychometric properties [20]. Response options are based on a 4-point Likert scale ranging from ‘none or almost none of the time’ to ‘all or almost all of the time’. We assumed correlated residuals between the positively worded items as reported by others [20].

Potential stressors affecting mental health, as proposed by Brooks et al. [16], were measured mostly with self-developed constructs as they were deemed the most adequate for the specific and novel context of the COVID-19 epidemic. These stressors were contextualized to the study population and to the exposure (i.e., a stay-at-home order vs. quarantine) as illustrated in Additional file 1. Level of boredom, inadequate supply of resources, perceived pandemic-related stigma and insufficient financial resources were measured by 1 item each. Fear of infection was measured by a 4-item construct. Tailored to the study population, two 4-item constructs were used to measure academic stress and institutional dissatisfaction. Responses were measured through 4, 5, and 11-point Likert scales ranging between 2 extremes, such as ‘strongly agree’ to ‘strongly disagree’. The duration of exposure to stay-at-home order was measured using the date of response submission. More detail on the measures can be found in Additional file 1 and in the study protocol [17].

### Data-analysis

Descriptive statistics were used to provide an overview of participants’ answers to essential sociodemographic variables, the outcome and the stressors.

Confirmatory factor analysis was used to assess the measurement models for depressive symptoms severity, academic stress, institutional dissatisfaction and fear of being infected. The objective of confirmatory factor analysis is to test whether data fit a hypothesized measurement model which assigns a value to an unobserved or latent variable based on its’ relationship with observed variables. A second-order model was used for fear of being infected since it included two subconstructs: infection of oneself and infection of someone in one’s personal network. The following indices of fit and related benchmarks for acceptable fit were used [21]: comparative fit index (CFI) (≥0.95), Tucker-Lewis index (TLI) (≥0.95), root mean square error of approximation (RMSEA) (≤0.08), and standardized root mean square residual (SRMR) (≤0.08). Diagonally weighted least squares was used for parameter estimation as latent constructs were assumed normally distributed.

To test the association between the hypothesized stressors and depressive symptoms severity, the following constructs were regressed on the CES-D scores: boredom, inadequate supply of resources, perceived pandemic-related stigma, insufficient financial resources, inadequate information, fear of infection, fear of significant others being infected, academic stress and institutional dissatisfaction. The following factors were used as control variables: gender, age, the number of people living with, citizenship, being in relationship, and financial ability to cover living expenses before the stay-at-home order. To test the hypothesized mediation through the stressors boredom and academic stress, a structural equation model was used with the CES-D as outcome and duration of the stay-at-home order as independent variable. Criteria for mediation were based on the definition by Cerin [22]: (1) the mediating variable relates to the independent variable; and (2) occurrence of a significant association between the mediating variable and the outcome variable, after adjustment for the independent variable. The fit of the mediation model was assessed based on the same fit indices and benchmarks used for the measurement models. Data were analyzed with R software and the packages ‘lavaan’ [23] and ‘semTools’ [24].

## Results

### Population characteristics

The study population’s mean age was 21 and included a higher proportion of females, bachelor students and natives (see Table 1). More than half of the participants reported to be in a steady relationship and the majority was living together with two or more people. More than 90% reported having sufficient resources to cover their living expenses.

**Table 1:** Summary characteristics and depressive symptoms scores of university students in Belgium, April–May 2020 (*N* = 18,301)

Characteristic	Category	proportion (%)
Age (years)	mean: 21.0 (SD: 2.32)	
	range: 17–30	
Gender	Female	73.9
	Male	26.1
Citizenship	Belgian	91.8
	Foreign	8.2
Study program	Bachelor	75.9
	Masters	24.1
First-year student		22.6
steady relationship		47.6
N° of people living with (during the stay-at-home order)	0	3.3
	1	11.6
	2 or more	85.1
Insufficient financial resources to cover living expenses (before the stay-at-home order)		3.1
CES-D 8 depressive symptoms scores	mean	10.8
	standard deviation	5.08

Legend: *CES-D* Center for Epidemiological Studies Depression Scale

On average, concern about being infected or severely ill was rated lower than concern about someone in the participants’ personal network being infected or severely ill (see Table 2). A majority of students reported sufficient financial resources to cover their expenses. Approximately one third reported to be bored most or all of the time. Ratings of the items related to academic stress indicated an increase of this stressor due to COVID-19 among a high proportion of students. Responses to items related to institutional dissatisfaction were mixed, with some items reflecting satisfaction and others dissatisfaction in a higher proportion of students.

**Table 2:** Descriptive statistics of stressors and related items among university students in Belgium, April–May 2020 (*N* = 18,301)

‘Stressor’ / related items	Category or scale	Statistic
**‘Fear of being infected’:**		mean (SD)
Concern of being infected	0 (not at all) -10 (very)	4.5 (2.7)
Concern of becoming severely ill because of COVID		4.5 (2.9)
Concern of infection in personal network		7.4 (2.2)
Concern of someone in personal network becoming severely ill because of COVID-19		7.8 (2.3)
**‘Concern of health services running out of supplies’**		5.1 (2.8)
		Prop. (%)
**‘level of boredom’**	none of the time	33.5
	some of the time	36.3
	most of the time	21.4
	all of the time	8.8
**‘COVID-19 related academic stress’:**		
Increase in academic workload	strongly agree	35.6
	agree	30.9
	neither agree nor disagree	18.2
	disagree	11.9
	strongly disagree	3.4
Course expectations are less clear	strongly agree	35.6
	agree	39.5
	neither agree nor disagree	11.9
	disagree	10.8
	strongly disagree	2.2
Interference with academic performance	strongly agree	38.2
	agree	30.5
	neither agree nor disagree	14.4
	disagree	13.1
	strongly disagree	3.9
Change in teaching methods causes distress	strongly agree	40.9
	agree	33.3
	neither agree nor disagree	12.9
	disagree	9.8
	strongly disagree	3.2
**‘COVID-19 related institutional (dis)satisfaction’:**		
Perceived decrease in quality of education	strongly agree	20.7
	agree	29.8
	neither agree nor disagree	28.3
	disagree	17.7
	strongly disagree	3.5
Sufficient information about changes due to COVID	strongly agree	11.0
	agree	41.2
	neither agree nor disagree	24.0
	disagree	16.7
	strongly disagree	5.2
Adequate implementation of protective measures	strongly agree	12.6
	agree	42.2
	neither agree nor disagree	27.5
	disagree	12.7
	strongly disagree	5.1
University staff is accessible to share concerns	strongly agree	6.8
	agree	25.5
	neither agree nor disagree	34.2
	disagree	21.7
	strongly disagree	11.8
**‘Sufficient financial resources to cover expenses’**	strongly agree	40.1
	agree	33.0
	neither agree nor disagree	12.5
	disagree	9.6
	strongly disagree	3.9
**‘The participant reported hiding symptoms from others’**		37.5

### Measurement models

Item indicators of the constructs measuring depressive symptoms severity, academic stress and institutional dissatisfaction were significant (z > 1.96) and loaded adequately on the latent variables (see Additional file 2). Criteria of fit were met for these constructs.

### Association between stressors and depressive symptoms severity

Each of the hypothesized stressors was positively associated with depressive symptoms severity independent of their adjustment for the control variables (see Table 3). Stressors associated with a higher score of depressive symptoms severity included ‘academic stress’, ‘institutional dissatisfaction’, ‘boredom’ and ‘fear of infection’ associated with, respectively, a 0.66, 0.51, 0.25, and 0.27 increase in depressive symptoms severity (on a scale from zero to three) per one-unit increase of the stressor. More response units were used to measure the stressors ‘insufficient medical supplies at health services’ and ‘fear of infection’ which implies that, in case of equal estimates, the effect size between two extreme response positions will be higher compared to stressors measured with less response units.

**Table 3:** Associations between stay-at-home order related stressors and depressive symptoms severity among university students in Belgium, April–May 2020 (*N* = 18,301)

variable	scale	Base model	Crude model	Adjusted model
		Estimate (SE)	Estimate (SE)	Estimate (SE)
age	yrs.	−0.030** (0.003)		−0.024**(0.003)
female gender	0–1	0.290** (0.016)		0.284** (0.016)
non-Belgian citizenship	0–1	0.148** (0.027)		0.009 (0.026)
having a steady relationship	0–1	−0.118** (0.015)		−0.119** (0.014)
living with 1 person^a^	0–1	−0.249** (0.044)		−0.219** (0.043)
living with 2 or more people^a^	0–1	−0.267** (0.040)		−0.217** (0.039)
Insufficient financial resources (before the stay-at-home order)	0–1	0.179** (0.009)		0.025* (0.011)
fear of infection	0–10		0.281** (0.013)	0.266** (0.014)
level of boredom	0–3		0.239** (0.007)	0.246** (0.007)
academic stress	0–4		0.655** (0.012)	0.663** (0.012)
insufficient medical supplies	0–10		0.055** (0.002)	0.049** (0.002)
institutional dissatisfaction	0–4		0.514** (0.014)	0.512** (0.014)
insufficient fin. Resources	0–4		0.151** (0.006)	0.144** (0.008)
perceived stigma^b^	0–1		0.140** (0.020)	0.118** (0.020)

Legend: unstandardized parameter estimates of the association between depressive symptoms severity as a latent construct and the control variables (base model), the hypothesized stressors without control variables (crude model) and the hypothesized stressors with control variables (adjusted model). Fear of infection, academic stress and institutional dissatisfaction represent latent constructs. Parameter estimates correspond to the effect on depressive severity on a scale from 0 to 3 (‘none of the time’ to ‘all of the time’) per 1-unit change of the predictor. ^a^ reference: living with no other people. ^b^ estimates of perceived stigma were based on a subsample of participants reporting symptoms that could potentially evoke stigma. *P*-value codes: *p* < 0.05 ‘*’,*p* < 0.001 ‘**’

All controlling variables showed a significant association with depressive symptoms severity in the base model. In the adjusted model, the association with non-Belgian citizenship and insufficient financial resources (before the stay-at-home order) disappeared or weakened, suggesting their association being explained by one of the stressors. Sensitivity analysis based on complete case analysis produced similar results.

### Mediation path model

**Fig. 1:**
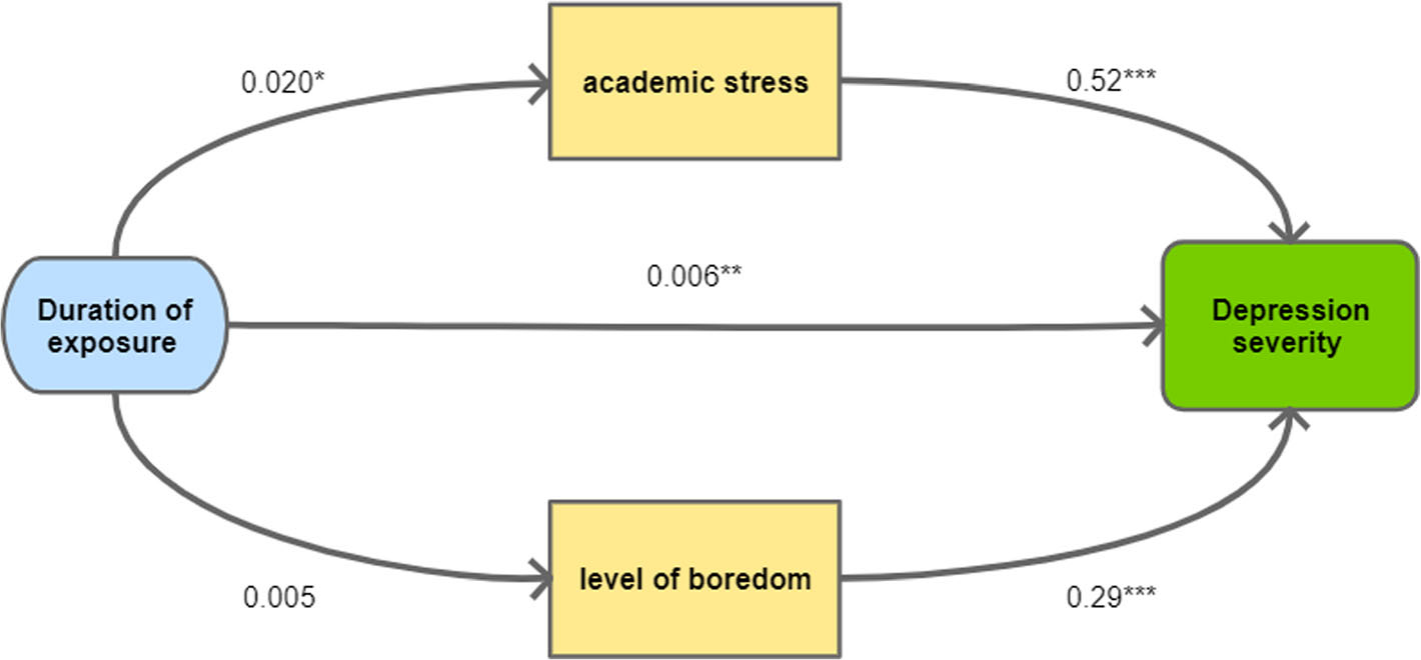
Mediation model between duration of exposure and depressive symptoms severity among university students in Belgium, April–May 2020 (*N* = 18,301). The estimates are fully standardized and adjusted for the control variables mentioned in methods. Duration of exposure was measured in days. *P*-value codes: *p* < 0.05 ‘*’, *p* < 0.01 ‘**’, *p* < 0.001 ‘***’

Per extra day of exposure to the stay-at-home order, average scores of depressive symptoms severity were 0.009 higher (i.e. the total association between duration of exposure and depressive symptoms severity). This association was partly mediated by academic stress, but not by perceived level of boredom (see Fig. 1). The model showed an acceptable fit (CFI=0.943; TLI=0.971: RMSEA=0.060; SRMR=0.056). Sensitivity analysis based on complete case analysis produced similar results.

### Discussion

This study provides novel insight into the repercussions of a stay-at-home order implemented during the peak of the COVID-19 pandemic on depressive symptoms severity among students attending higher education in Belgium. The aim of this study was to test a contextualized version of the stressors that Brooks et al. identified [16] in specific populations exposed to quarantine. Our findings indicated that those stressors were also relevant in students exposed to a stay-at-home order. The stressors ‘academic stress’, ‘institutional dissatisfaction’, and ‘fear of infection’ were associated with a strong increase in depressive symptoms severity. Duration of the exposure also showed to have an effect on depressive symptoms severity, which was mediated by ‘academic stress’. Being in a steady relationship and living together with others were associated with a lower depressive symptoms severity.

To our knowledge, academic stress and institutional dissatisfaction have not been tested as stressors in a similar setting study during the COVID-19 pandemic. The strong association with depressive symptoms severity is in line with studies during a non-epidemic setting showing depression to be highly correlated with perceived study demands [8]. With regards to academic stress, a majority of students indicated an increase in academic workload, course expectations being less clear, being concerned about their academic performance and being distressed because of a change in teaching methods. Regarding institutional dissatisfaction, half of the students reported a decrease in quality of education and one third reported university staff (e.g., a professor, a student counselor, etc) not being accessible to share concerns about COVID-19. However, more than half of the students also reported that their HEI provided sufficient information about changes due to COVID-19 and adequate implementation of protective measures. Overall, these findings are important warning signs for HEI to carefully monitor and address academic demand and for academic staff to assure the quality of teaching and a student-centered approach.

On average, fear of being infected was rated lower than fear of someone from the participants’ personal network being infected. This suggests that students are more concerned about significant others than about themselves being infected, which we assume may be due to seeing themselves running a lower risk of complications because of their younger age. Evidence from other studies has been mixed regarding these stressors. Cao et al. found having significant others to be infected by COVID-19 an important risk factor for experienced anxiety among students, potentially due to a higher perceived probability of themselves being infected [14]. However, the latter assumption is in contrast with our findings. A study in a general population in Italy found an effect on depression if an acquaintance was infected by COVID− 19, but not if a family member was infected by COVID− 19 [25].

Living together with others and being in a steady relationship were associated with lower depressive symptoms severity scores. We assume this is due to social interaction preventing depressive tendencies through boredom or feelings of loneliness, but they may also relate to social support when one goes through personal challenges or academic stress. Similar associations have been shown in other studies [8], including a study during the COVID-19 pandemic [14].

Perceived stigma among people with COVID-19 related symptoms did not show a strong association with depressive symptoms, while it was found a major theme among people being quarantined [16]. We assume this can be explained by the difference between quarantining and a stay-at-home order, with the latter being applicable to the whole population and not only to a subgroup of infected people. In addition, our study only considered people who were not diagnosed with COVID-19.

Duration of exposure to the stay-at-home order only had a small effect, which was expected since data-collection only started at day 39 of the stay-at-home order. Moreover, the implemented stay-at-home order rather was a light version with, for instance, outdoor physical activity still allowed. The few studies examining the duration of exposure in quarantined individuals [16], reported an effect on other mental health outcomes, but not on depression. The model in our study was in support of the association between duration and depressive symptoms severity being partly mediated by academic stress. Again, this emphasizes the need for monitoring and addressing students concerns and frustrations.

Two of the major strengths of this study include: (1) the large sample of students from 14 HEIs in the height of the Belgian COVID-19 crisis; and (2) data covering the full range of mental health stressors outlined by a recent review study on the mental health consequences of quarantine. This study also has several limitations. Putting our findings into perspective, the big sample size of the study increased the probability of a type-1 error. The stay-at-home order also needs to be seen as a ‘light version’ with several movements still allowed (e.g., outdoor physical activity, commuting, public transport). We also want to remark that it is impossible to know to which extent the associations found in our study can be attributed to the stay-at-home order, direct effects of the pandemic (e.g., mortality), or other causes. For example, the established association with ‘fear of being infected’ likely depends more on direct COVID-19 related morbidity and mortality than on the stay-at-home order triggered by the pandemic. Another limitation of the study was the use of an online survey which is prone to self-selection bias. Comparing our study sample with governmental data of higher education students enrolled in Flanders in 2018–2019 [26], reveals a higher proportion of females (+/− 20%) and a similar proportion of foreign nationals. We did not see a decline in values of key variables such as depressive outcome, perceived academic stress and institutional dissatisfaction among students who participated during a later stage of the study, which is in support of sample validity for these key variables. We conclude that based on this convenience sample, we cannot claim estimates of depression status and other variables to be representative of the overall Belgian student population. We think, however, that after controlling for key variables, the reported relationships are reasonable reflections of a general trend. Another limitation concerns the adequacy of the measures being used. Because of a lack of contextually validated constructs – the novelty and specific character of the COVID-19 epidemic forced us to be innovative – most of these measures were developed and used for the first time with several one or two-item constructs. Finally, the cross-sectional study design did not allow us to examine any longitudinal effects or confirm causal relationships.

## Conclusions

Our findings underline the importance of a student-centered approach in students exposed to a stay-at-home order during the COVID-19 pandemic. We recommend authorities and HEI to carefully consider whether and if yes, for how long and how strict a stay-at-home order is essential using a multidisciplinary approach. In case such an intervention is required, major stressors such as ‘academic stress’ and ‘institutional dissatisfaction’ need to be monitored and addressed by HEI. Concretely, we recommend clear and straightforward communication and to stimulate interactions between students and teachers through real-time teaching. Students may also be encouraged to ensure in-person social interactions (e.g., temporarily move in with parents, family or friends), which were suggested protective. Monitoring and reconsidering academic demand is warranted. We further recommend HEI to monitor students’ mental well-being and to offer accessible mental health support, with special attention for students who are prone to social deprivation and who are living alone. Finally, our findings confirm the importance of further research on mental health during pandemic containment measures.

## Supplementary Information

**Additional file 1.** Contextualisation of COVID-19 stressors.

**Additional file 2.**

## Data Availability

The data underlying this article will be shared on reasonable request to the corresponding author.

## Abbreviations

CES-D: Center for Epidemiological Studies Depression
CFI: Comparative Fit Index
COVID-19: Coronavirus Disease of 2019
HEI: Higher Education Institutions
TLI: Tucker-Lewis Index
RMSEA: Root Mean Square Error of Approximation
SRMR: Standardized Root Mean square Residual
