# Supplementary material for: Disentangling depression in Belgian higher education students amidst the first COVID-19 lockdown (April-May 2020)
Source: Arch Public Health. 2021 Jan 7;79:3. doi: 10.1186/s13690-020-00522-y (PMC7789891; doi:10.1186/s13690-020-00522-y)
Supplement: Supplementary file 1 — Additional file 1. Contextualisation of COVID-19 stressors. [file 13690_2020_522_MOESM1_ESM.docx]

| **Stressors by Brooks et al.** | **Contextualized Stressors*** | **Questionnaire items** | **Answer scale** |
| --- | --- | --- | --- |
| Fears of infection (fears about their own health or fears of infecting others) | Fear of being infected and fear about the participants’ health. | How worried are you to get infected by COVID-19? | 0 (not at all) - 10 (very) |
|  |  | How worried are you that you will get severely ill from a COVID-19 infection? | 0 (not at all) - 10 (very) |
|  |  | How worried are you that anyone from your personal network will get infected with COVID-19? | 0 (not at all) - 10 (very) |
|  |  | How worried are you that anyone from your personal network will get severely ill from a COVID-19 infection? | 0 (not at all) - 10 (very) |
| Level of boredom | Level of boredom | How much of the time during the past week were you bored? | 0 (None of the time) - 3 (all of the time) |
| Level of frustration | Academic stress | The university/college provides poorer quality of education during the COVID-19 outbreak as before. | 0 (strongly disagree) - 4 (to strongly agree) |
|  |  | The university/college has sufficiently informed me about the changes that were implemented due to the COVID-19 outbreak. | 0 (strongly agree) - 4 (to strongly disagree) |
|  |  | I am satisfied with the way my university/college has implemented protective measures concerning the COVID-19 outbreak. | 0 (strongly agree) - 4 (to strongly disagree) |
|  |  | I feel I can talk to a member of the university/college staff (e.g., professor, student counsellor) about my concerns due to the COVID-19 outbreak. | 0 (strongly agree) - 4 (to strongly disagree) |
| Inadequate basic supplies (specifically, supplies from public health authorities to people in a quarantine setting) | Inadequate medical supplies at health services | How worried are you that doctors and hospitals will not have sufficient medical supplies to handle the COVID-19 outbreak? | 0 (not at all) - 10 (very) |
| Inadequate information (specifically from public health authorities) | Institutional dissatisfaction | My university/college workload has significantly increased since the COVID-19 outbreak. | 0 (strongly disagree) - 4 (to strongly agree) |
|  |  | I know less about what is expected of me in the different course modules/units since the COVID-19 outbreak. | 0 (strongly disagree) - 4 (to strongly agree) |
|  |  | I am concerned that I will not be able to successfully complete the academic year due to the COVID-19 outbreak. | 0 (strongly disagree) - 4 (to strongly agree) |
|  |  | The change in teaching methods resulting from the COVID-19 outbreak has caused me significant stress. | 0 (strongly disagree) - 4 (to strongly agree) |
| Financial loss and socio-economic distress | Insufficient financial resources | 'I had sufficient financial resources to cover my monthly costs during the COVID-19 outbreak' | 0 (strongly agree) - 4 (to strongly disagree) |
| Perceived stigma | Perceived stigma | Were there occasions that you tried to hide symptoms such as coughing, sneezing, or a runny nose from other people? | 0 (no) – 1 (yes) |

* Contextualized to the study population (i.e. higher education students) and to the exposure (i.e. a stay-at-home order versus quarantine)
