# Supplementary material for: Disentangling depression in Belgian higher education students amidst the first COVID-19 lockdown (April-May 2020)
Source: Arch Public Health. 2021 Jan 7;79:3. doi: 10.1186/s13690-020-00522-y (PMC7789891; doi:10.1186/s13690-020-00522-y)
Supplement: Supplementary file 2 — Additional file 2. [file 13690_2020_522_MOESM2_ESM.docx]

**Specification and evaluation of the measurement models of the latent variables.**

| **latent variable:** | **CES-D** |  |  |  |  |
| --- | --- | --- | --- | --- | --- |
| Chi-square statistic | 1997.495 |  |  |  |  |
| Degrees of freedom | 19 |  |  |  |  |
| P-value (Chi-square) | 0.000 |  |  |  |  |
| CFI | 0.987 |  |  |  |  |
| TLI | 0.981 |  |  |  |  |
| RMSEA | 0.075 |  |  |  |  |
| 90 % CI RMSEA | 0.073 | 0.078 |  |  |  |
| SRMR | 0.032 |  |  |  |  |
|  |  |  |  |  |  |
| indicators: | Estimate | Std.Err | z-value | p-value | Std.all |
| item 1 | 1.000 |  |  |  | 0.896 |
| item 2 | 0.817 | 0.005 | 151.550 | 0.000 | 0.732 |
| item 3 | 0.605 | 0.007 | 88.040 | 0.000 | 0.542 |
| item 4 | 0.814 | 0.006 | 146.941 | 0.000 | 0.730 |
| item 5 | 0.770 | 0.006 | 139.085 | 0.000 | 0.690 |
| item 6 | 0.656 | 0.007 | 97.755 | 0.000 | 0.588 |
| item 7 | 0.941 | 0.005 | 202.996 | 0.000 | 0.843 |
| item 8 | 0.675 | 0.006 | 106.170 | 0.000 | 0.605 |
|  |  |  |  |  |  |
| Covariances: | Estimate | Std.Err | z-value | p-value | Std.all |
| item 4 ~~ item 6 | 0.291 | 0.005 | 55.017 | 0.000 | 0.526 |
|  |  |  |  |  |  |
| **latent variable:** | **fear of infection** |  |  |  |  |
| Chi-square statistic | 273.342 |  |  |  |  |
| Degrees of freedom | 0 |  |  |  |  |
| P-value (Chi-square) | 0.000 |  |  |  |  |
| CFI* | NA |  |  |  |  |
| TLI* | NA |  |  |  |  |
| RMSEA* | NA |  |  |  |  |
| 90 % CI RMSEA* | NA |  |  |  |  |
| SRMR* | NA |  |  |  |  |
|  |  |  |  |  |  |
| indicators | Estimate | Std.Err | z-value | p-value | Std.all |
| subfactor 1 | 1.000 | NA | NA | NA | 1.007 |
| subfactor 2 | 0.732 | NA | NA | NA | 0.693 |
|  |  |  |  |  |  |
| subfactor 1: |  |  |  |  |  |
| item 1 | 1.000 | NA | NA | NA | 0.869 |
| item 2 | 0.908 | NA | NA | NA | 0.789 |
|  |  |  |  |  |  |
| subfactor 2: |  |  |  |  |  |
| item 3 | 1.000 | NA | NA | NA | 0.925 |
| item 4 | 0.979 | NA | NA | NA | 0.905 |
|  |  |  |  |  |  |
|  |  |  |  |  |  |
| **latent variable:** | **academic stress** |  |  |  |  |
| Chi-square statistic | 173.794 |  |  |  |  |
| Degrees of freedom | 2 |  |  |  |  |
| P-value (Chi-square) | 0.000 |  |  |  |  |
| CFI | 0.996 |  |  |  |  |
| TLI | 0.988 |  |  |  |  |
| RMSEA | 0.069 |  |  |  |  |
| 90 % CI RMSEA | 0.060 | 0.077 |  |  |  |
| SRMR | 0.016 |  |  |  |  |
|  |  |  |  |  |  |
|  | Estimate | Std.Err | z-value | p-value | Std.all |
| item 1 | 1.000 |  |  |  | 0.690 |
| item 2 | 0.949 | 0.010 | 92.061 | 0.000 | 0.655 |
| item 3 | 1.146 | 0.010 | 110.316 | 0.000 | 0.791 |
| item 4 | 1.200 | 0.011 | 108.666 | 0.000 | 0.828 |
|  |  |  |  |  |  |
|  |  |  |  |  |  |
| **latent variable:** | **institutional dissatisfaction** | |  |  |  |
| Chi-square statistic | 105.262 |  |  |  |  |
| Degrees of freedom | 2 |  |  |  |  |
| P-value (Chi-square) | 0.000 |  |  |  |  |
|  |  |  |  |  |  |
| CFI | 0.996 |  |  |  |  |
| TLI | 0.987 |  |  |  |  |
| RMSEA | 0.053 |  |  |  |  |
| 90 % CI RMSEA | 0.045 | 0.062 |  |  |  |
| SRMR | 0.015 |  |  |  |  |
|  |  |  |  |  |  |
|  | Estimate | Std.Err | z-value | p-value | Std.all |
| item 1 | 1.000 | 0.552 |  |  | 0.552 |
| item 2 | 1.278 | 0.017 | 73.299 | 0.000 | 0.705 |
| item 3 | 1.425 | 0.020 | 70.926 | 0.000 | 0.786 |
| item 4 | 0.885 | 0.015 | 57.818 | 0.000 | 0.488 |

Factor loadings are reported as unstandardized parameters (estimate) with their standard errors (std.Err), related z-values and p-values and as standardized parameters (Std.all). * this model was not identified. CES-D = Center for Epidemiological Studies Depression Scale. CFI = comparative fit index; TLI = Tucker-Lewis index; RMSEA = root mean square error of approximation; 90% CI = 90% confidence interval for RMSEA; SRMR = standardized root mean square residual.
